# Detection of a Novel African Swine Fever Virus with Three Large-Fragment Deletions in Genome, China

**DOI:** 10.1128/spectrum.02155-22

**Published:** 2022-08-24

**Authors:** Yankuo Sun, Zhiying Xu, Han Gao, Sijia Xu, Jing Liu, Jiabao Xing, Qiyuan Kuang, Yang Chen, Heng Wang, Guihong Zhang

**Affiliations:** a African Swine Fever Regional Laboratory of China (Guangzhou), Guangzhou, China; b Key Laboratory of Zoonosis Prevention and Control of Guangdong Province, College of Veterinary Medicine, South China Agricultural University, Guangzhou, China; c Guangdong Laboratory for Lingnan Modern Agriculture, Guangzhou, China; d National Engineering Research Center for Breeding Swine Industry, South China Agricultural University, Guangzhou, China; Changchun Veterinary Research Institute

**Keywords:** African swine fever virus, large-fragment deletions, genomes

## Abstract

We reported a novel African swine fever virus (ASFV) strain that had a three-large-fragment deletion and unique variations in genome. This isolate displayed a nonhemadsorbing phenotype and had homogeneous proliferation compared with the wild-type ASFV strain. Our findings highlighted the urgent need for further investigation of ASFV variations in China.

**IMPORTANCE** African swine fever virus (ASFV) has been circulating in China for 5 years, and low virulent strains with changes in the genome have been reported. Nevertheless, there is still a lack of knowledge about the epidemic strains at the whole-genome level. This study reported a novel strain and further analyzed its genomic and biological characteristics. In addition, our study also suggests that whole-genome sequencing plays a key role in the epidemiology investigation of ASFV variations.

## OBSERVATION

African swine fever (ASF) was first reported in China in August 2018 (1). Since then, the African swine fever virus (ASFV) has rapidly spread nationwide, causing severe and continuous economic losses to the Chinese pig industry (2). With its spread and evolution in China, naturally attenuated ASFV isolates with genome variations have emerged (3). Our study described the biological and genome characteristics of the isolate obtained from clinical specimens in the field.

**FIG 1:**
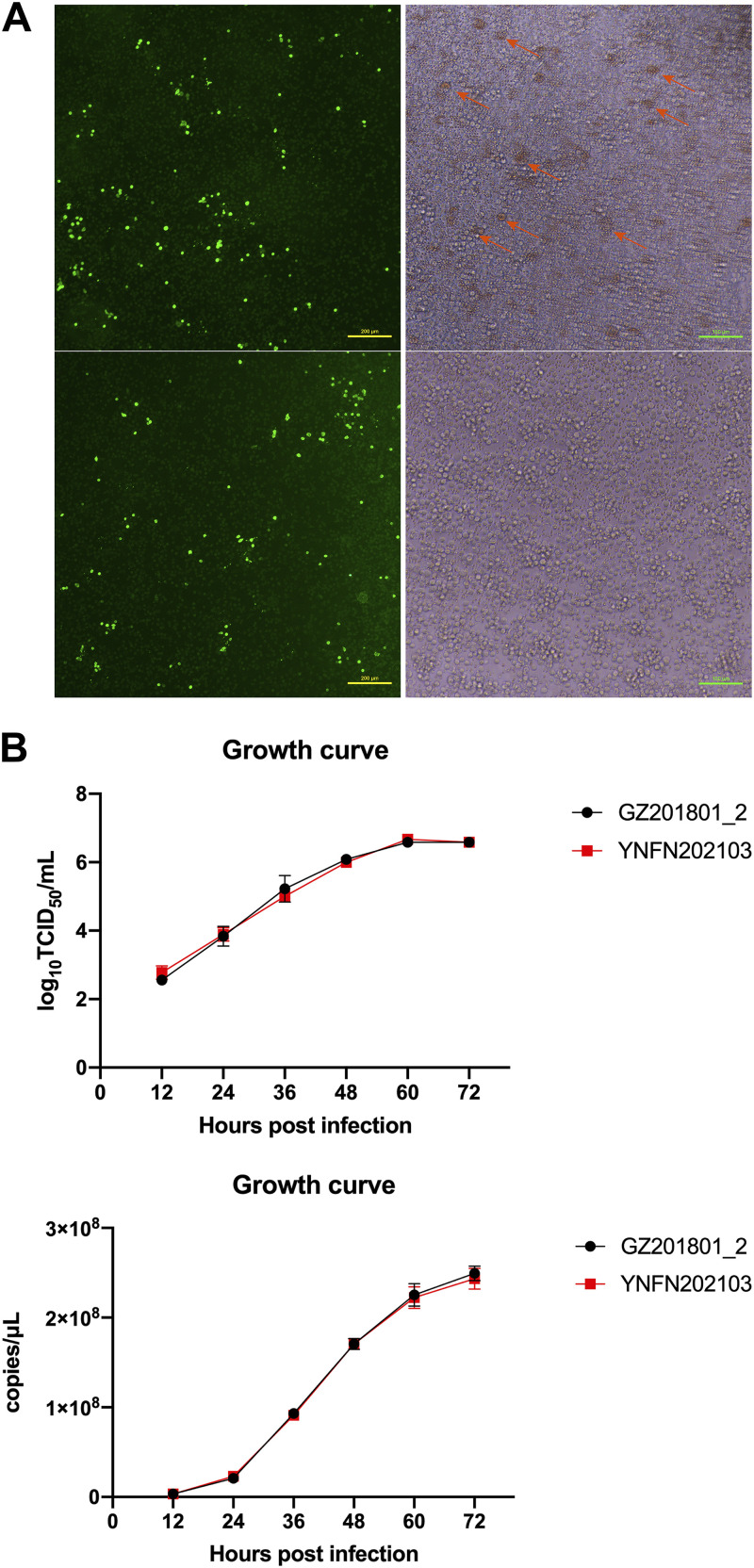
(A) Biological characteristics of ASFV_YNFN202103 were elucidated through hemadsorption and immunofluorescence (stained with anti-p30 monoclonal antibody) assays, and (B) its growth curve (MOI = 0.1).

We collected serum and tissues samples from pigs suspected to be ASFV-positive in one herd from the Yunnan province during our routine genome-wide and nationwide monitoring of circulating ASFVs in China. After background investigation, the pigs were reported to present relatively atypical clinical symptoms, including fever, skin cyanosis, depression, and joint swelling. Moreover, the whole herd showed very low mortality (~20%). Our diagnostic lab confirmed this case as ASFV positive using a real-time PCR (RT-PCR) identification kit targeting the *B646L* gene (Beijing MingRiDa Science and Technology Development Co., Ltd., catalog number 010688870). All the live virus manipulation of ASFV in this study was carried out in a biosafety level-3 laboratory at South China Agricultural University (Guangzhou, China). The serum was processed, and the virus was isolated using porcine alveolar macrophages (PAMs). Virus isolation was confirmed via an immunofluorescence assay, and the new isolate was named ASFV_YNFN202103 (GenBank ID: ON400500) (Fig. 1A). The biological characteristics of ASFV_YNFN202103 were examined *in vitro*, and a hemadsorption (HAD) assay was first performed (4). Compared with wild-type ASFV_GZ201801_2 (ON263123), which is a highly pathogenic genotype II strain isolated from an early outbreak in South China, ASFV_YNFN202103 displayed a loss of hemadsorption ability due to the deletion of the *EP402R* gene (CD2v protein), while ASFV_GZ201801_2 showed a typical HAD phenotype (Fig. 1A). To assess the dynamic growth of the isolate, PAMs were infected at a multiplicity of infection of 0.1, and cell supernatants were subsequently collected and processed in a time course RT-PCR/TCID_50_ assay, postinfection. The growth curves showed that ASFV_YNFN202103 exhibited a proliferation similar to that of wild-type ASFV_GZ201801_2 (Fig. 1B).

**FIG 2:**
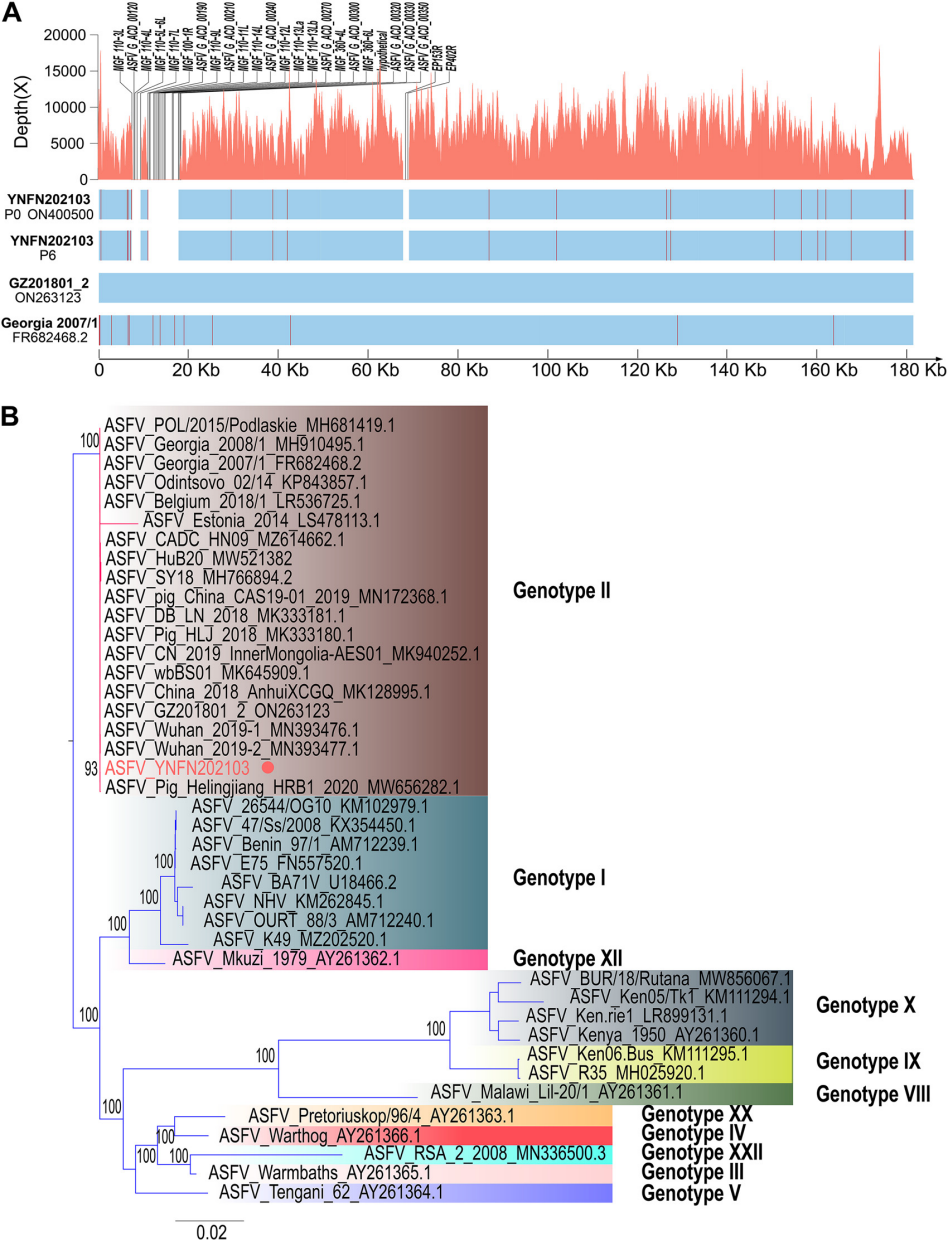
**(**A) Depth and coverage of ASFV_YNFN202103 were calculated from its sequencing data using SAMtools v1.10 (6). Specific characteristics are marked on the top. General comparison between ASFV_YNFN202103 and wild-type ASFV_GZ201801_2 (ON623123), and between ASFV_YNFN202103 P6 and ASFV_Georgia 2007/1 (FR682468.2) are presented on the bottom (gaps have been blanked, and indels/substitutions have been marked in red). (B) Phylogenetic analysis based on the full-length genome of representative ASFV isolates was performed using the ggtree v3.0.4 (ASFV_YNFN202103 has been marked with a red dot) (15).

Viral DNA was extracted from the serum, and the whole-genome sequencing of ASFV_YNFN202103 was performed using the MGI next-generation platform (BGI, Shenzhen), based on the workflow previously described (5). Briefly, raw sequencing data showing ~100% coverage and at least 100× depth were assembled using the MEGAHIT v1.2.9 (6). All reads were mapped to ASFV_GZ201801_2 to find the indels and substitutions using BWA v0.7.17 (7). Primers targeting specific regions were designed, and the corresponding amplicons were processed via Sanger sequencing for proofreading to exclude gaps and variant sites caused by sequencing errors (Table 2 in the supplemental material). Although gene deletion events occurring in the ASFV genome are common (8), the verified full-length genome of ASFV_YNFN202103, after proofreading, showed a series of unique characteristics compared to wild-type ASFV_GZ201801_2. The most striking feature was a three-large-fragment deletion that covered 23 genes, accounted for over 10,000 bp in length, and was mainly located in the multigene family 100, 110, and 360 regions and the *EP153R/EP402R* genes (Fig. 2A). These three-large-fragment deletions consisted of 2,114 bp, 7,772 bp, and 1,477 bp fragments, successively placed from 5′ to 3′ (supplemental Table 1). Specific nucleotide variations were also analyzed. Among the 29 indels and substitutions, only two were synonymous mutations, indicating a highly adaptive evolution to the environment. Interestingly, very few studies have reported site mutations in the *B646L* gene, which encodes the p72 capsid protein, and that was used as the genotyping gene for ASFV (9, 10). Its genome showed one nonsynonymous substitution (Ser31Phe). Among the 29 gene variations, 13 caused single amino-acid substitutions, while the others caused greater changes in the genome, including frameshifting and premature termination, which may result in remarkable differences between phenotypes (supplemental Table 2). Furthermore, ASFV_YNFN202103 was processed for six serial passages to verify whether specific genomic variations affected the stability of the viral genome. The 6th passage was sequenced using the MGI NGS platform. Compared with those of ASFV_GZ201801_2 and ASFV_Georgia 2007/1 (FR682468.2), the sequence of the 6th passage of ASFV_YNFN202103 showed no distinct genetic variation, illustrating its good genomic stability (Fig. 2A). Furthermore, according to our phylogenetic analysis, ASFV_YNFN202103 belonged to genotype II, too (Fig. 2B).

Since ASFV was first isolated in China in 2018, not only wild-type, but also several naturally gene-deleted virus strains have been detected in ASF outbreaks (3, 11). Because our experimental conditions are limited, the pathogenicity of ASFV_YNFN202103, with a unique three-large-fragment deletion and 29 nucleotide indels/substitutions, was further needed to be verified by animal pathogenicity experiments. Previous studies had found that deletions in the ASFV have been linked to the adaptation of viruses during cell culture (Vero and HEK293T) (12, 13), while our genome was obtained directly from clinical specimens. Many of these gene-deleted strains are displayed as viral attenuates since the infected herds showed milder symptoms and much lower mortality than the wild type (3), some of which have been used to rationally attenuate vaccine candidates. *EP402R* and *EP153R* deletions with no genetic markers were reported early in 2020 in China (3), and the deletion patterns were totally different from the reported strains. The emergence of various gene-deleted isolates reveals the complexity of the ASFV circulating in China, and the identification of this isolate brings more challenges to the study of ASFV epidemiology in China and ASFV diagnosis.

The biological characteristics of ASFV_YNFN202103 may offer deeper insights into the functions of those genes that are absent or mutated here but are present in wild-type ASFV_GZ201801_2, as one or several of these absent or mutated genes might be the critical factor(s) defining the virulence of ASFV strains in China, which requires further research (14). Moreover, the viral genomic stability was confirmed through six serial passages, as the comparison between P0 and P6 showed 100% homogeneity.

In summary, we reported a novel genotype II ASFV in the field with a distinguished three-large-fragment deletion and 29 indels/substitutions throughout its genome. The total of these deletion regions was over 10,000 bp, and the deletion pattern has never been reported before. Combined with the biological characteristics in this study, the strong replication capability and stable genome of this virus could become a potentially epidemic strain. Long-term ASFV genomic surveillance with continuously optimized strategies is essential to combat new variants of concern in China.

### Data availability.

The raw sequencing data generated from clinical samples (P0) and cell supernatants collected after six successive passages (P6) have been deposited in the NCBI Sequence Read Archive (SRA) database under the BioProject accession number PRJNA847162. The genome sequence of ASFV_YNFN202103 generated in this study have also been deposited in GenBank and assigned accession number ON400500.
